# Analysis of the Emails From the Dutch Web-Based Intervention “Alcohol de Baas”: Assessment of Early Indications of Drop-Out in an Online Alcohol Abuse Intervention

**DOI:** 10.3389/fpsyt.2021.575931

**Published:** 2021-12-15

**Authors:** Wouter A. C. Smink, Anneke M. Sools, Marloes G. Postel, Erik Tjong Kim Sang, Auke Elfrink, Lukas B. Libbertz-Mohr, Bernard P. Veldkamp, Gerben J. Westerhof

**Affiliations:** ^1^Department of Psychology, Health Technology, University of Twente, Enschede, Netherlands; ^2^Department of Research Methodology, Measurement Data Analysis, University of Twente, Enschede, Netherlands; ^3^Tactus Addiction Treatment, Enschede, Netherlands; ^4^Netherlands eScience Center (NWO), Amsterdam, Netherlands

**Keywords:** therapeutic change process research (TCPR), alcohol use disorder (AUD), drop-out, web-based psychotherapeutic interventions, e-mail data, machine learning

## Abstract

Nowadays, traditional forms of psychotherapy are increasingly complemented by online interactions between client and counselor. In (some) web-based psychotherapeutic interventions, meetings are exclusively online through asynchronous messages. As the active ingredients of therapy are included in the exchange of several emails, this verbal exchange contains a wealth of information about the psychotherapeutic change process. Unfortunately, drop-out-related issues are exacerbated online. We employed several machine learning models to find (early) signs of drop-out in the email data from the “Alcohol de Baas” intervention by Tactus. Our analyses indicate that the email texts contain information about drop-out, but as drop-out is a multidimensional construct, it remains a complex task to accurately predict who will drop out. Nevertheless, by taking this approach, we present insight into the possibilities of working with email data and present some preliminary findings (which stress the importance of a good working alliance between client and counselor, distinguish between formal and informal language, and highlight the importance of Tactus' internet forum).

## Introduction

Addictive behaviors and substance dependencies have a global impact, with alcohol use disorder as the prevailing substance abuse disorder (1). It is estimated that, around the world, 283 million individuals suffer from alcohol use disorder, representing ~5.1% of all adults (2). As these numbers are predicted to increase globally (3), the need for accessible treatment becomes more apparent than ever. Yet, there is large delay between onset of the disorder and first treatment contact (4). A growing number resort to online solutions for their drinking problems (5, 6). Although web-based psychotherapeutic interventions have been established as effective interventions for alcohol use disorder (7, 8), they are plagued by high rates of drop-out (9, 10), thereby adding to the already well-known problems of high drop-out in alcohol treatment (11). The aim of this study is to analyze whether emails that were written by clients early in the treatment process can predict drop-out of the online treatment.

Some *general* advantages of web-based interventions are that they have a lower threshold for first treatment contact (12, 13), they can be as effective as traditional face-to-face therapy (14–16), they come at a low cost (17), and they have usually no or only short waiting lists (12). Online, many clients feel they can maintain their privacy (18), feel less stigmatized (19, 20), and (sometimes even) prefer the impersonal nature of the web, as they do not have to disclose their feelings and problems in person (21). Online interventions for substance dependencies form a large part of the online offer, with many targeting alcohol use disorder specifically (22).

The *specific* advantage of web-based interventions for alcohol use disorder is the all-time availability. Websites can be accessed every hour of the day and every day of the year, which is a great advantage over face-to-face treatment for those who cannot attend to treatment at business hours (23). This is of special importance when treating alcohol use disorder (5), as the willingness of clients to change their drinking behaviors is often of volatile nature and easily affected by (negative) events, which can also occur during holidays (24). Even though web-based interventions make it difficult for a counselor to react to the non-verbal cues of clients, they are a helpful and welcome addition to the treatment of alcohol use disorder.

Yet, there is no debate about the biggest drawback of web-based interventions (25, 26): they are plagued by a high rate of drop-out, on average ~50% (9), and for some, even as high as 99% (26, 27). The same problem is known for online alternatives for alcohol use disorder specifically (9, 11, 28). Postel (29), for example, reported a drop-out rate of 54%, whereas 84.5% dropped out in the study of Linke et al. (30).

We did not find studies that set out to specifically address the reasons for dropping out of an online intervention for alcohol use disorder (if reported, drop-out analyses usually are one of many complementary analyses). Drop-out is referred to in the literature as *pre-mature termination, non-usage, low attrition*, or *retention* (10, 31, 32). Studies that analyze drop-out often use different sample groups, diffuse treatments, and diverse subtypes of disorders (33), which makes it even more difficult to compare drop-out between studies. How drop-out should be defined is also not universally agreed upon: some argue that only those who did not finish the complete intervention dropped out; others argue that clients who did not reach a certain cap of required attended sessions should be considered drop-out (34), and some say that only the judgment of the counselor can determine who dropped out, as it is also possible that a client dropped out because he or she already experienced the beneficial effects of therapy (35, 36). These distinctions matter, as the different approaches affect the drop-out rates reported (37). In line with Eysenbach's *Law of Attrition*, in this study, participants are considered drop-out when they did not finish all the treatment sessions that required to complete the treatment protocol (38).

Knowing who is likely to complete—and thereby hopefully benefit from—an intervention provides a better basis for an evidence-based allocation of clients to treatment (39). Therapy change process research is the field that has addressed these kinds of questions (40). Online interventions have the advantage that interactions between clients and counselors are saved over time, for example, in emails that they exchange. The abundance of data these create allows for innovative methods like text mining (41). In the current study, we will use two basic text mining approaches to learn more about their use in predicting drop-out in an online intervention for alcohol use disorder.

Given high rates of drop-out, also in this intervention, the aim of this study is to assess whether the first mails of clients include any early “warning” signs of drop-out. To the best of our knowledge, this has not been done before and we did not find applications of text mining applications specifically tailored to study drop-out for alcohol use disorder.

## Method

First, we will use a bottom-up approach by “simply” counting the most frequently used words in emails (42), and second, we will also use a top-down approach, building on dictionary-based approaches in psychology (43). The dictionary-based program *Linguistic Inquiry and Word Count* program [LIWC; (44)] has perhaps the most widespread use in (general) psychological research and is available in many languages, including Dutch (45, 46). LIWC allows one to analyze many aspects of the email texts, using both linguistic markers like function words and punctuation and psychological markers like affect words, cognitive processes, and personal concerns (47). Liehr et al. (48) provide an example in which they assessed self-change by applying LIWC to written stories and studied stressful feelings over the course of an intervention for substance dependencies.

We will use an evidence-based intervention for treating alcohol use disorder: the web-based intervention “*Alcohol de Baas*,” loosely translated from *Dutch* as “Look at your drinking” (19, 49). AdB is rooted in cognitive behavioral therapy and motivational interviewing, both empirically substantiated approaches for the treatment of substance dependencies (50, 51). The intervention consists of two parts: the first focuses on drinking habits, the second on behavior change. In the first part, counselors support clients in analyzing their drinking habits through several assignments and assessments that are followed up by feedback from the counselor. It ends with a personalized advice to the client.

The second part focuses on changing the drinking habits of clients and aims to replace the thoughts associated with alcohol cravings by more helpful ones. After about 10 weeks, the intervention ends with the formulation of an action plan for maintaining the new drinking behavior or sobriety to prevent relapse. Postel (29) demonstrated that the intervention led to a significant decrease in alcohol consumption, which was maintained at a 6-month follow-up. The intervention attracted almost 1,000 users per year, a substantial interest given the size of the Dutch population. Clients and counselors primarily used email to establish the beneficial effects of the intervention, so important aspects of the therapeutic process should be included in these emails.

### Study Design

The current study uses a naturalistic, prospective design with consecutive clients who signed up for the online intervention *Alcohol de Baas* (AdB 29). The data includes personal characteristics, the first four emails written by clients, and information about treatment drop-out.

Postel et al. (19) received ethical approval for (re-)analysis of AdB. Prior to starting treatment, participants gave their informed consent that their data could be (re-)used, but had the right to withdraw at any moment.

### Participants

**Figure 1:**
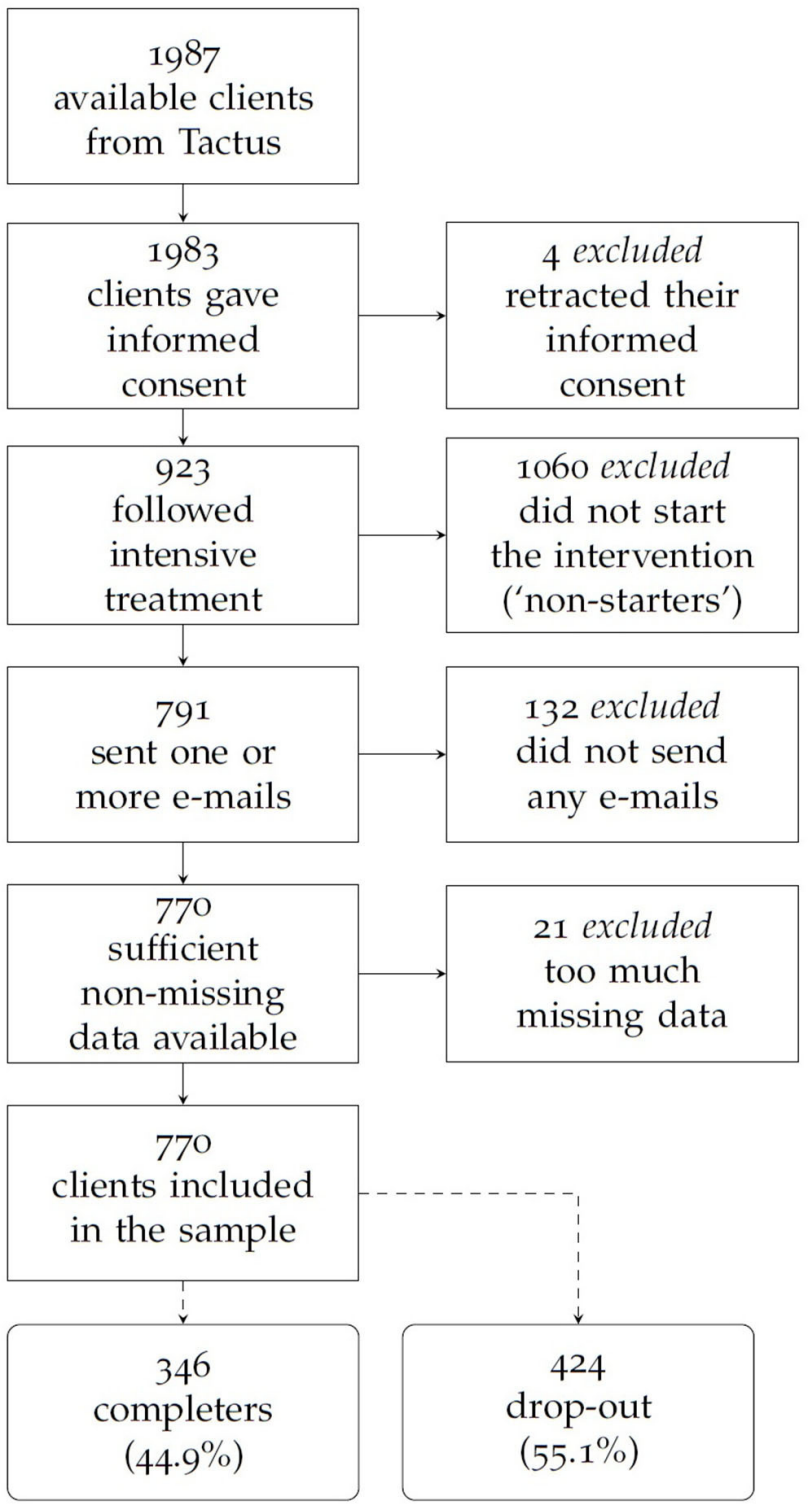
Flowchart of the (excluded) clients.

Visitors who are concerned about their own drinking patterns had direct access to the webpages of AdB and can register for the program online. All participants who registered themselves for AdB with self-reported alcohol problems had to be over 16 years old, which was the legal drinking age in the Netherlands when the study was conducted. Of the 1,987 consecutive persons who registered up to 2017, 4 were excluded because they retracted their informed consent, 1,060 did not start the intervention, 132 did not send any emails, and 21 had too much missing data (see Figure 1). Hence, 770 participants were included in the current study. Tables 1, 2 provide an overview of their personal characteristics. Their median age is 46 years (range between 17 and 78 years). The majority were female, of Dutch nationality (and spoke Dutch), were married and finished a higher vocational degree. They smoked occasionally, but did not use drugs, nor did they gamble. Their main reason to start with treatment was that they worried about their drinking behavior: the median consumed units of alcohol (10 g of ethanol) of alcohol per week was 36 at onset of the program. About half of the sample frequently experienced feelings of depression or other psychological problems (24). From 770 clients, 346 completed the full treatment, resulting in a drop-out rate of 55.1%. For a more in-depth characterization of clients, we refer the interested reader to the four case descriptions provided in the Appendix in Supplementary Material.

**Table 1:** Overview of the client's age, years of problematic alcohol consumption, and the average units of consumed alcohol.

	**Drop-out**				**Completer**			
	***M***	***SD***	**Min**.	**Max**.	***M***	***SD***	**Min**.	**Max**.
Age	44.4	11.0	17	78	47.7	10.2	19	75
Cons. years	17.9	10.5	3	35	19.3	11.5	5	40
Av. cons. alc.	8.1	7.0	0	25	7.0	5.0	0	24

**Table 2:** Overview of the demographic characteristics in-take questionnaire, split to drop-out and completer.

**Variables**	**Drop-out**		**Completer**	
	**(*****N*** **=** **424)**		**(*****N*** **=** **346)**	
	***N***	**%**	***N***	**%**
**Gender**				
Males	209	61.1	133	38.9
Females	215	50.2	213	49.8
**Nationality**				
Dutch	22	44.9	27	55.1
No answer	402	55.8	319	44.2
**Education**				
Primary	5	55.6	4	44.4
Lower vocational education	69	63.3	40	36.7
School of higher general secondary education/pre-university education	56	62.9	33	37.1
Intermediate vocational education	103	60.2	68	39.8
Higher vocational education	137	52.5	124	47.5
University	40	40.8	58	59.2
No answer	14	42.4	19	57.6
**Ever followed treatment before**				
Yes	25	48.1	27	51.9
No	308	56.2	240	43.8
No answer	91	53.5	79	46.5
**Reason starting the intervention**				
I think I am drinking too much	334	55.9	264	44.1
I want advice about my alcohol consumption	14	58.3	10	41.7
Something happened and I want to change my drinking behavior	36	53.7	31	46.3
Others think I am drinking too much	14	70.0	6	30.0
Other reasons	24	45.3	29	54.7
No answer	2	25.0	6	75.0
**Tobacco use**				
Never	164	46.7	187	53.3
Now and then	36	60.0	24	40.0
Daily	224	62.4	135	37.6

### Instruments

Personal characteristics of clients were assessed before the intervention started for a complete overview of the variables used (see Tables 1, 2).

Available text data consisted of emails that the clients and their counselors exchanged during the intervention AdB. Depending on the part of the program, the emails were more or less tailored to each individual client. We only considered texts of clients containing 20 words or more. The mean number of emails written by clients was 20.8, with a maximum number of 116 emails. The current study focused on the first four emails written by clients as an early indicator for drop-out.

Clients were labeled as a “completer” when they received an email with the word *afsluiting* (Dutch for *closure*). These emails were inspected to make sure that they were indeed related to a completed treatment which, for example, included finishing the final assignment *actieplan* (“action plan” in *Dutch*). All clients who did not qualify for these criteria were labeled as “treatment drop-out.” The labeling of emails was conducted by Van den Hazel (52).

### Data Processing

The first step in the analysis is the *preprocessing* of the data using Python, an interpreted, high-level, and general-purpose programming language (53). The goal is to have the data in a format suitable for further data analysis. First, some client emails included texts from a previous email by the counselor. As these quotes were not written by the client, we removed these from earlier emails. Next, we normalized all texts by converting all capitals to lowercase characters. We then divided the text into tokens and sentences with *Frog* (54) and NLTK (55). NLTK tallies sentences by counting word-terminal end-of-sentence punctuations like the period, question mark, and exclamation mark. NLTK has a list of abbreviations, which are not included in the punctuation and sentence count. Word-internal punctuation, like the first period in “e.g.,” is ignored. Handling of interjections depends on their punctuation, for example, “Oh?” is a separate sentence while “Oh,” is part of the following sentence. Sentence fragments and quotes with end-of-sentence punctuation are counted as separate sentences.

The next step is to *anonymize* the emails by replacing the names, dates, numbers, locations, medical problems, and other (“miscellaneous”) entities with the abbreviations “PER,” “DATE,” “NUM,” “PRO,” and “MISC,” respectively (56). We used the *Frog*-program for *named entity recognition* and for anonymization of these entities (54). For example, a first client email started as: “Dear [PER], my name is [PER].” Because named entity recognition is a machine learning task, the anonymization procedure was not without flaws, for example, because entities were misspelled. To ensure that all personal information was removed, we checked the analyses of Frog repeatedly and adjusted the anonymization and pre-processing accordingly.

After pre-processing and anonymization, word use was investigated using *n*-grams. *N*-grams are sequences of *n* consecutive words occurring in the text. More specifically, we employed unigrams, the simplest of the *n*-grams, as it counts the frequencies of the individual words in the text. Next, we analyzed the content of the emails with the *Linguistic Inquiry and Word Count* [LIWC; (44)]. We used the Dutch translations of LIWC (45, 46). LIWC consists of several dictionaries with subcategories (57, 58). For example, *positive emotion words* is a subcategory of the dictionary *affective processes* and consists of words like *happy, pretty*, and *good*. LIWC counts the percentage of words in an email number belonging to a specific subcategory. For each email, the output contained 76 variables. Besides the score per email, we calculated the average across the four emails for each client for each of these 76 variables. The repeated measures consisted out of 76 × 4 LIWC variables, whereas the *averages* consisted of 76 LIWC variables.

### Statistical Analysis

All analyses were performed in Python. We included all personal variables in a first step (see Tables 1, 2). In a second step, we included all text variables to see what could be gained from the text analyses over and above the personal variables. In the second step, we did one set of analyses with the LIWC variables for the first four emails as *repeated measures*, and another set with *averages* across these four emails.

We employed three types of statistical models: a logistic regression, a neural network, and decision trees. For all logistic regression models, our dependent variable was drop-out (yes/no), whereas we used all personal characteristics and all LIWC categories as independent variables. A random (or “naive”) distribution of clients would result in a correct classification of ~50%, because both groups (roughly) had an equal number of observations. To have an impression of the “baseline” performance of statistical models, we first conducted a (standard) logistic regression. The training method we used for the logistic regression was *mini-batch gradient descent*, with the *binary cross-entropy* as the loss function.

Neural networks are well-known for their predictive accuracy on complex tasks (59, 60) and are often applied in text mining (13, 61–64). We used a multi-layer perceptron, with five fully connected layers. The repeated measures used a slightly different architecture: the first layer of the network only takes the personal data as input, and the LIWC scores only enter the network in the second layer. Maity and Pal (65) showed that this architecture could improve results when dealing with repeated measures data.

Decision trees are well-known for the insightful “decision-maps”, which are relatively straightforward to interpret (66–68). Ensemble methods are a class of decision tree models that have better predictive performance (62), and these so-called boosting methods combine several “weak” classifiers to improve prediction of the final boosted classifier (69). We applied XGBoost (70), a (boosted) decision tree that—for many tasks—is known to outperform standard tree-based models (13). For the repeated measures, we also used two Mixed Effect Random Forests (MERF; “longitudinal decision trees”). A MERF enhances the standard decision tree by including mixed (or “random”) effects, which can lead to substantial performance improvements when dealing with clustered data (71). To our understanding, the MERF software of Hajjem et al. (71) does not include the option to assess both clustered structures simultaneously. Therefore, our first MERF used the repeatedly observed LIWC scores as clusters, and as a result, the MERF model took the longitudinal structure of the data into account. The second MERF used the clients as clusters.

In order to adequately assess model performance while still maintaining an acceptable training–test-set ratio, we used five-fold cross-validation for each model. We will only discuss test-set performance (the confusion matrices in the next section only report the test-set numbers). We reported the precision, recall, accuracy and the *F*_1_-score. We assign the most weight to the first two scores, as they balance the other two (and some other aspects of correct and incorrect classifications).

## Results

We first studied the most frequently used words in the emails of the drop-out and the completers [see Table 3 for an overview of (the translations of) these words]. Table 4 contains the performance metrics of the models that we employed and also contains the “naive” baseline model based on random chance. As can be seen in Table 4, the unigram model does not outperform baseline classification. This means that none of the words in Table 3 is able to discriminate between the drop-out and completers. However, some word classes in Table 3 stand out as they could indicate some potentially relevant differences between the drop-out and completers: we discuss these three word classes in the next section.

**Table 3:** Top ten most commonly used words in the e-mails for those who completed the intervention, and for those who dropped out.

**Completers**		**Drop-Out**	
**English**	***Dutch***	**English**	***Dutch***
that	*die*	you (*formal*)	*u* ^1^
was	*was*	have	*heb*
he	*hij*	your (*formal*)	*uw* ^1^
felt	*voelde*	Dear (*formal*)	*Beste*
glass	*glas*	detox	*detox*
also	*ook*	general practitioner	*huisarts* ^2^
counselor	*therapeut* ^2^	are	*zijn*
cancer	*kanker*	kind	*vriendelijke*
it	*het*	use	*gebruik*
pain	*pijn*	Your (*plural*)	*jullie*
forum	*forum* ^3^	regards	*groet*

*This Table presents the results of the unigram model; we discuss the observations numbered 1–3 in the results section*.

**Table 4:** The performance metrics of the models.

**Model**	**Accuracy**	**Precision**	**Recall**	***F_**1**_*-score**
**Baseline**				
Negative only	0.551	0	0	0
Positive only	0.449	0.449	1	0.620
0.449 change of positive	0.505	0.449	0.449	0.449
***N*** **-grams**				
Unigrams	0.591	0.538	0.633	0.582
**Average**				
Logistic regression	0.560	0.509	0.322	0.372
MLP	0.575	0.568	0.272	0.346
Decision tree	0.610	0.562	0.638	0.594
**Demographic only**				
Logistic regression	0.571	0.525	0.218	0.286
MLP	0.570	0.525	0.293	0.356
Decision tree	0.587	0.540	0.570	0.551
**LIWC only**				
Logistic regression	0.534	0.381	0.158	0.222
MLP	0.529	0.458	0.208	0.283
Decision tree	0.579	0.523	0.717	0.604
**Repeated measures**				
Logistic regression	0.595	0.533	0.721	0.609
MLP	0.525	0.522	0.302	0.374
Decision tree	0.612	0.548	0.799	0.648
Advanced NN	0.566	0.519	0.537	0.522
MERF timing	0.541	0.533	0.474	0.501
MERF client	0.525	0.514	0.471	0.491

### Word Use

Our first observation involves the usage of informal pronouns; in Dutch, there is a formal and informal way for addressing others. Three of the most frequently used words by the clients who dropped out the treatment were formal (see number 1 in Table 3), addressing the counselor in a somewhat remote manner (“*I don't know what you can do for me, aside from forwarding my file*”). Aside from the “distance” between the client and counselor, formal language is also used to express some sort of misunderstanding (“*Did I understand you correctly, you want me to answer all the questions? That will take ridiculously long for you to read*”).

Second, the clients who completed the intervention often refer to a *psychotherapist*, whereas the ones who dropped out frequently mention a *general practitioner* (see number 2 in Table 3). It appears that the first—in addition to the counselor from Tactus—also have a psychotherapist (“*Yes, I really believe I need a therapist with whom I can fight about my ideas and thoughts*”). This therapist is often perceived as a source of support (“*My therapist agrees that I could benefit from these situations as well*”), and it appears that the therapist often discusses topics that are similar to the ones in the Tactus intervention (“*I discussed this yesterday as well with my therapist*”). The clients who dropped out on the other hand often mention their general practitioner, to whom the Tactus counselors refer excessive drinkers (“*I went to my general practitioner, and my blood pressure was good*”). The general practitioner of some appears to be aware of the alcohol dependency (“*My general practitioner knows about my alcohol abuse*”), whereas this is not the case for others (“*I tried to discuss this with my general practitioner, but I was shocked by the reaction I got*”).

Thirdly, the clients who completed the intervention mention a *forum*. In addition to the AdB program, Tactus also offers access to an online internet forum where it is possible to discuss and meet with other participants of the program. According to Postel (29), the forum receives great user satisfaction, and offers support, motivation, and engagement (p. 136). It appears that the clients who dropped out do not use this forum, as they do not mention it.

### LIWC Analyses

The results of the LIWC averages can be found in Table 4 (performance metrics) and 5 (confusion matrix). Table 4 does not indicate that any analyses based on the LIWC averages outperforms naive classification: the accuracy and *F*_1_-score rarely exceed the random baseline classification. For the LIWC averages, the *F*_1_-scores are low for the logistic regression and the multilayered perceptron (MLP in Table 4), mainly due to poor recall. The decision tree performed slightly better, with a higher accuracy and a recall that is substantially higher than for the other two models.

The results for the repeated measures are displayed in Table 4. As the confusion matrices of the LIWC repeated measure analyses were similar to Table 5, we did not include these here for the sake of brevity. Table 4 indicates that model performance remains similar to the naive classification. The performance of the longitudinal decision tree and MERF are on par with the neural network of the LIWC averages. Even though we included all LIWC categories and personal characteristics as input in our analyses, Table 4 does not indicate that the longitudinal models are (better) capable of predicting drop-out. Given that we employed a wide array of models, we conclude that there are no “large,” “powerful,” or “strong” predictors of drop-out in the first four emails.

**Table 5:** Confusion matrix for the models using the averaged LIWC scores.

**Model**		**Observed**	
		**+**	**–**
Logistic regression	+	44	34
	–	26	29
MLP	+	55	39
	–	15	24
Decision tree	+	53	43
	–	17	20

*A completer is labeled with a “+,” drop-out is labeled with a “–.” Only the test-set (N = 134) has been included*.

## Discussion

Web-based psychotherapy is an established alternative to classic face-to-face therapy, with the large drawback that almost all online interventions are plagued by a high rate of drop-out. In our data, we found a drop-out rate of nearly half, which was high, but similar to past studies (9). So, *why did these clients drop-out*? We tried to answer this question by comparing the first four emails of clients who completed the intervention to those of clients who dropped out. We used a wide array of models, but could not associate the email texts to drop-out.

### Word Use

The analysis of word use showed that there are some differences in the first four emails between clients who did or did not complete the intervention. The (*Dutch*) words “*u*” and “*uw*,” indicating polite and formal ways to address a counselor, were used with greater frequency by those who dropped out. This could point to differences in the therapeutic alliance between client and counselor (72). The fact that the completers do not address their counselors in a formal way could be an indication that they feel a stronger therapeutic alliance (73). Establishing openness and trust requires effort which is perhaps difficult to do online for those who dropped out. It is possible that clients who already know “how to work with a counselor” could be further in their process of becoming less dependent on alcohol.

There is also a difference between the usage of the words *therapist* by clients who completed and *general practitioner* by those who dropped out. For some, the intervention could be a recommendation from their general practitioner, while others could have found the intervention on their own, suggesting a difference in extrinsic or intrinsic motivation. Another interpretation is that those who dropped out could perceive their alcohol usage as a medical problem, whereas those who completed could perceive their drinking behavior as a psychological problem and thereby be more open for the psychological support the intervention offers (74). Some clients might perceive medical care as the only form of “real” healthcare, not expressing much faith in psychological counseling and thereby investing less in the therapeutic alliance.

The last finding is that the forum was mentioned only by those who completed the intervention. The forum could be a great source of support for them, whereas for those who dropped out, this could indicate less engagement with the intervention. It could also be that those who actively participate in the forum are further in their own psychotherapeutic process, as they know it also takes some effort from themselves to become less dependent on alcohol. It would therefore not be far-fetched to recommend that the intervention could try to establish more tie-ins with the forum (and *vice versa*).

### LIWC Analysis

LIWC is an often used program that could be helpful in determining textual aspects of therapy that are relevant for drop-out. However, we were unable to achieve satisfactory predictive accuracy based on LIWC features, even though different statistical models were used. Perhaps LIWC is too “crude” to pick up the nuances that are present in these kinds of text data. For example “*I was so angry when I was a child*” is equally counted to the category anger as “*I am so angry right now*,” even though the first sentence describes the past and this might have changed by now. Also, “*I hate my family*” and “*I love my family*” both contain words for the LIWC category family, yet have an entirely different emotional connotation. So the category *family* in this example does not allow for a meaningful differentiation between these two statements.

LIWC has been developed with a broad purpose and not specifically for the problem addressed in this study. Although LIWC is a popular tool among psychologists, our study contributes to a more nuanced understanding of LIWC. Other researchers did show the potential of creating more fine-tuned dictionaries for alcohol use disorder (75) or suggested to combine several LIWC categories (76). Perhaps dictionaries that target nuances that are specifically tailored to alcohol use disorder will be more helpful in understanding drop-out. Our list of most frequently used words could be helpful as a first step.

Dutch is a relatively small language with ~24 million speakers, and LIWC is the best and only readily available alternative at the moment. There might be other relevant dictionaries in English or another language. However, simply translating such dictionaries through standard available translation software is arguably naive, as it could result in a loss of linguistic and cultural nuances.

### Strengths and Limitations

To the best of our knowledge, there are no earlier studies that explore drop-out for alcohol use disorder through a text-mining approach. Having a better understanding of how (and why) drop-out occurs does not only have scientific value, it can also greatly benefit the clinical practice, for example, making counselors aware of the word use of their clients that might be related to drop-out. As we present one of the first attempts to systematically study emails for alcohol use disorder, the combination of bottom-up analyses of word use and top-down dictionaries like the LIWC seemed well-suited for the purpose at hand. Starting analyses in an earlier phase would be an important recommendation to detect possible problems for analyses such as clients including quotes from counselors in their emails and to check the feasibility of programs used.

The measure for drop-out was in a sense rather crude as it is a complex and multidimensional construct. Clients could drop out for several reasons: they might not like the intervention, be hesitant to build a therapeutic relation, experience a crisis or even quit the intervention because they already experienced benefits. A recommendation for future research is therefore to include the nuances of drop-out. TPCR-related studies would benefit from *structured* datasets that include more labeling from counselors and clients, so that it becomes possible to conduct a more nuanced analysis of the text constructs. This could decrease the time spent on pre-processing, while the value of the data analysis increases greatly.

Although from a pragmatic viewpoint the dataset is quite large, especially in the Netherlands, the dataset was relatively small for machine learning approaches. Including more data points, like more emails of clients as well as emails of counselors, might improve the performance of the models. There is a need for further developments as more email data becomes available, a complete “manual” study becomes too labor intensive (77). Developing systems that are more suitable for the export of the large numbers of (relevant) texts from emails is important for numerous reasons. As of yet, more often than not, this requires some familiarity with programming, which is not part of the standard curriculum of psychologists trained at the graduate level.

Ever since Sigmund Freud introduced the talking cure, conversation is the cornerstone to most forms of psychotherapy. Given the central position of the therapeutic exchange, a careful assessment of the therapeutic language could provide insight into what is happening in therapy. As AdB primarily relies on the exchange of emails between client and counselor, information about the therapeutic processes (drop-out included) should be present in these emails. Even though we were not able to produce models that were helpful in discriminating between the drop-out and completers, the qualitative interpretation of our findings does suggest that the emails contain relevant information.

## Data Availability Statement

The dataset presented in this article is not available: European Privacy regulation prohibits data sharing that can make human subjects identifiable. Inquiries about the datasets can be directed to g.j.westerhof@utwente.nl.

## Ethics Statement

Ethical review and approval was not required for this study, because we reused an existing dataset in accordance with the local legislation and institutional requirements. The participants provided their written informed consent to participate in the original study, which included later reuse of their data.

## Author Contributions

WS wrote the manuscript and supervised AE for his bachelor thesis and LL-M for his master thesis. ET conducted the data handling in Python and pre-processed the data. MP contributed the data and gave feedback throughout the process. AS helped WS with the supervision of AE and LL-M and gave, together with BV and GW, feedback throughout the process. BV and GW revised the manuscript for review. The data-analyses of AE formed the basis of the results-section, the literature review of LL-M contributed greatly to the introduction and discussion. All authors contributed to the article and approved the submitted version.

## Funding

This manuscript is the result of the *What Works When for Whom* project, which was supported by the Life Science eHealth domain of the Accelerating Scientific Discovery (ASDI) call from the Netherlands eScience Center (NLeSC; Amsterdam, the Netherlands): Grant No. 027.015.G04 awarded to AS. The NLeSC is the national knowledge center for the development and application of research software to advance scientific research, and was funded by the Netherlands Organization for Scientific Research (in *Dutch*: *Nederlandse organisatie voor Wetenschappelijk Onderzoek*; *NWO*) and SURF (abbreviation for *Samenwerkende Universitaire Rekenfaciliteiten* in *Dutch*).

## Conflict of Interest

The authors declare that the research was conducted in the absence of any commercial or financial relationships that could be construed as a potential conflict of interest.

## Publisher's Note

All claims expressed in this article are solely those of the authors and do not necessarily represent those of their affiliated organizations, or those of the publisher, the editors and the reviewers. Any product that may be evaluated in this article, or claim that may be made by its manufacturer, is not guaranteed or endorsed by the publisher.
