# Supplementary material for: Analysis of the Emails From the Dutch Web-Based Intervention “Alcohol de Baas”: Assessment of Early Indications of Drop-Out in an Online Alcohol Abuse Intervention
Source: Front Psychiatry. 2021 Dec 15;12:575931. doi: 10.3389/fpsyt.2021.575931 (PMC8714780; doi:10.3389/fpsyt.2021.575931)
Supplement: Supplementary file 1 [file Data_Sheet_1.docx]

# Appendix: Case descriptions

To further characterize the clients that were included in our sample, we give four anonymised case descriptions. All case descriptions rely on the information that respondents reported about themselves on the assessment questionnaire, and include some of the things they wrote about themselves in the e-mails. The case descriptions come from Giesler ([2019](#_bookmark34)) and Krstić ([2019](#_bookmark49)). Note that, even though certain aspects of the e-mails were anonymised (such as the number of consumed units of alcohol), the information that we report in the Tables and case descriptions were also available in the numerical data.

***Case 1.*** A 38 year old female, who regularly consumed alcohol for over ten years. At the onset of the treatment, she consumed 62 units of alcohol per week. Her goal of participating was to reduce alcohol intake to prevent health problems from reoccurring, and to make progress in her studies. The main motivation for drinking includes its rewarding and relaxing effect. Previous drinking habits have caused her loss of memory, missing lectures, self-sabotaging patterns (in terms of diet), headaches, and sleeping problems. An important factor for this client is also her husband, with whom she drinks on many occasions. The client noticed that she had problems quitting even when others around her were not drinking.

***Case 2.*** A 49 year old male, living with his wife and two children. The level of consumption at the beginning of the treatment included between 20 and 50 units per week. The client set the goal of reducing consumption, but emphasized the social and practical factors that justified the drinking behaviour. The client reported several physical problems including memory loss, tiredness, decrease in sexual arousal, and depression. His wife is an important factor during the treatment, and is described as a “*regular drinker*”. During the treatment, mood swings were dominant. Alcohol was important for the job of the client, as it was an important contributor to closing (business) deals. Mood swings were also frequently reported in the e-mails, emphasizing that alcohol is perceived as a reward.

***Case 3.*** Client is a 27 year married female, who got pregnant during the treatment.

The client had a medical history of potential eating disorders (she reported binge eating, dieting, and excessive exercises). Physical symptoms include fatigue, headaches, and sleep deprivation. At the beginning of the treatment, the client consumed 32 units of alcohol per week. Alcohol is perceived as a rewarding, socializing, and self-empowering factor. Due to her working environment, the client feels that she cannot deny drinks that are offered to her. On an emotional level, the client referred to disappointment and frustration, which are related to her self-esteem problems that are also reflected in her problems with body-image and previous binge eating episodes. For this client, establishing control over her symptoms are of great importance. Due to pregnancy, the client decided to alter her goal from reduction to complete abstinence.

***Case 4.*** A 48 year old male, living alone and has a conflictual relationship with his sister. His mother is in a nursing home, and he often interacts only with one friend. The initial consumption included 105 units per week. The client reported having a breakdown, due to which he consumed blood pressure and cholesterol medication. The most dominant emotions in the e-mails were anxiety, frustration, and overall dissatisfaction with his relationships, work, and life. The client showed indications of suicidal ideation and is often apologetic towards the counsellor. He perceived alcohol as a social factor, highlighting that “*there is no life or parties without alcohol*.”

**References**

Giesler L. Referential Activity in the E-Therapy Program “Look at Your Drinking” –A Text-

Mining Approach. (2019). Unpublished bachelorthesis: University of Twente (Enschede, The Netherlands).

Krstić M. Reading between the lines: Detecting emotion-abstract language use in the web-

based treatment “Look at your drinking” (2019). Unpublished masterthesis: University of Twente (Enschede, The Netherlands).
